# Serum NT-proBNP Concentrations in Pediatric Patients with a History of Idiopathic Cardiac Arrhythmias: A Pilot Study

**DOI:** 10.3390/ijms27198559

**Published:** 2026-09-24

**Authors:** Ewa Moric-Janiszewska, Joanna Wawszczyk, Małgorzata Kapral

**Affiliations:** Department of Biochemistry, Faculty of Pharmaceutical Sciences in Sosnowiec, Medical University of Silesia in Katowice, Jedności 8A, 41-200 Sosnowiec, Poland; jwawszczyk@sum.edu.pl (J.W.); mkapral@sum.edu.pl (M.K.)

**Keywords:** NT-proBNP, natriuretic peptides, idiopathic cardiac arrhythmias, antiarrhythmic therapy, pediatric cardiology, supraventricular tachycardia

## Abstract

Diagnosis and management of pediatric cardiac arrhythmias is challenging due to heterogeneous clinical presentation and limited availability of validated biomarkers. N-terminal pro-B-type natriuretic peptide (NT-proBNP) is a well-established marker of myocardial stress; its role in pediatric arrhythmias has not been fully elucidated. This pilot study included 26 children and adolescents with a history of idiopathic ventricular arrhythmias (VAs, n = 17) or supraventricular tachycardia (SVT, n = 9) and 20 hospital-based controls without arrhythmias. Serum NT-proBNP was measured by ELISA. Group differences were tested with the Mann–Whitney U test and correlations with Spearman’s coefficient. NT-proBNP concentrations were significantly higher in pediatric patients with a history of cardiac arrhythmias than in controls, with the difference remaining statistically significant for the VAs subgroup. The treated patients had significantly lower levels than the untreated individuals. NT-proBNP showed no significant associations with age, sex, echocardiography, or other laboratory parameters. In the VAs subgroup, NT-proBNP was moderately negatively correlated with baseline QRS duration. Given a limited patient population, these findings represent a pilot exploratory analysis. NT-proBNP should be considered a supportive biomarker reflecting functional myocardial strain rather than a stand-alone diagnostic tool for pediatric arrhythmia classification.

## 1. Introduction

Cardiac arrhythmias represent a significant challenge in pediatric cardiology. Although life-threatening cardiovascular pathologies are viewed primarily as an adult burden, they nonetheless account for approximately 5% of pediatric fatalities, usually resulting from sudden, fatal arrhythmias or severe hemodynamic disturbances [1,2,3,4,5,6]. Cardiac rhythm abnormalities are relatively prevalent in the pediatric population. They are frequently benign and asymptomatic, which often delays or complicates timely diagnosis [7,8,9]. Pharmacological therapy is essential in managing cardiac arrhythmias to significantly reduce the risk of severe cardiovascular complications, including sudden cardiac death (SCD) [10,11,12].

Cardiac arrhythmias are broadly categorized into supraventricular tachyarrhythmias (SVTs) and ventricular tachyarrhythmias (VTs), distinct in both their clinical presentation and underlying mechanisms [13,14]. SVTs, including atrial fibrillation and atrioventricular nodal re-entrant tachycardia (AVNRT), typically manifest with palpitations, dizziness, and hemodynamic stability or mild compromise, primarily driven by re-entry circuits or abnormal automaticity within or above the His bundle [13]. In contrast, VTs often arise from ischemic scars or structural heart disease through re-entry or triggered activity, presenting as severe hemodynamic instability, syncope, or sudden cardiac death [14]. Crucially, both arrhythmia types induce significant myocardial stress, high intraventricular pressure, and wall stretch, which serve as a potent trigger for the synthesis and release of N-terminal pro-B-type natriuretic peptide (NT-proBNP) [15]. Due to its longer half-life and greater plasma stability compared with BNP, NT-proBNP has become a widely used biomarker of myocardial strain [16,17,18,19,20,21].

**Figure 1:**
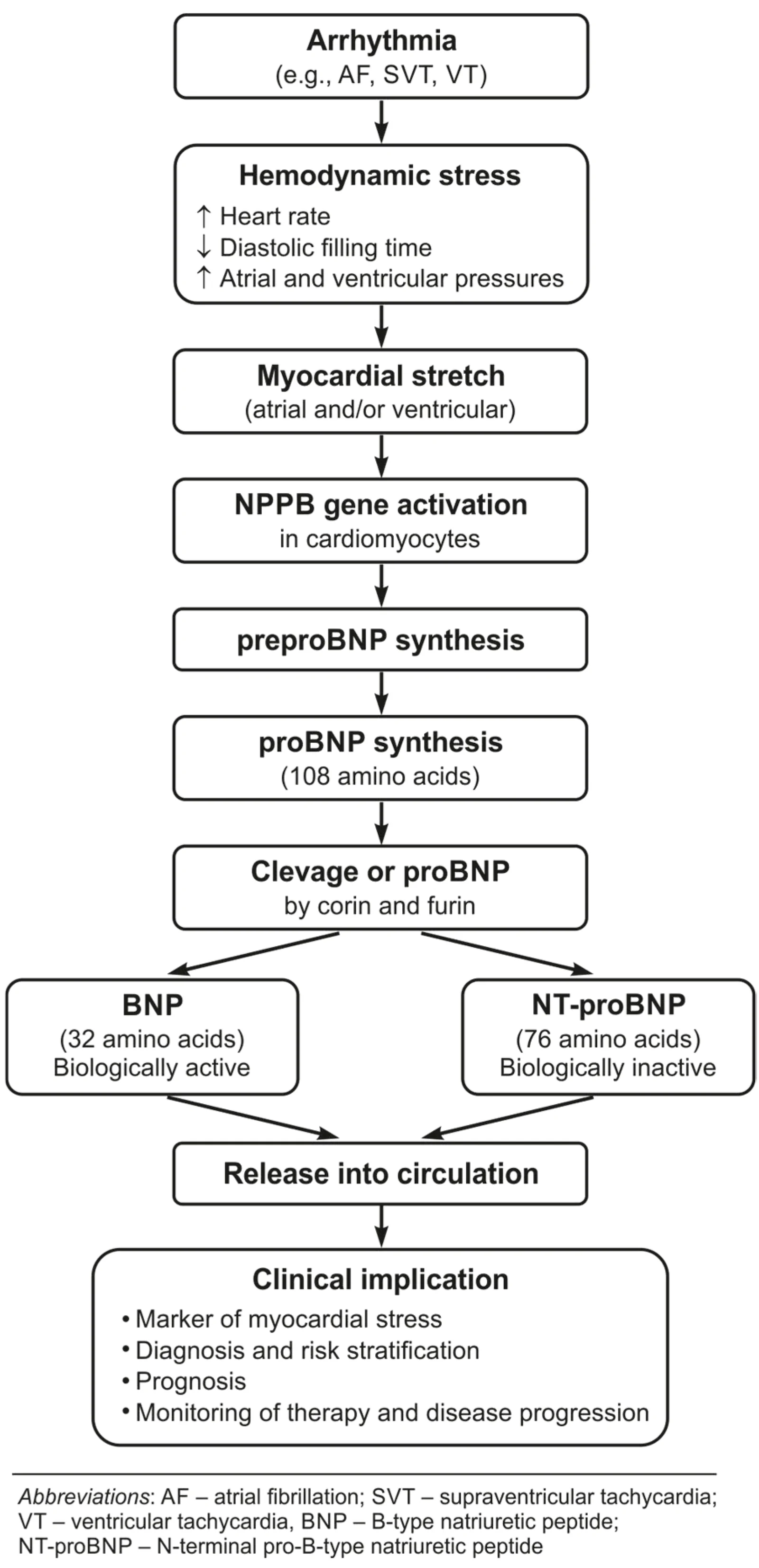
Mechanism of NT-proBNP release in arrhythmia [16,17,18,19,20,21,22,23,24,25,26,27].

Elevated circulating concentrations of BNP and its N-terminal fragment, NT-proBNP, serve as vital clinical biomarkers for heart failure and myocardial stress. In patients with arrhythmias, elevated NT-proBNP concentrations result from this immediate wall tension, exacerbated by the activation of the sympathetic nervous system, the renin–angiotensin–aldosterone (RAA) system, and secondary microvascular myocardial ischemia [16,17]. Elevated levels are directly correlated with the extent of myocardial remodeling, arrhythmia burden, risk of recurrence, and adverse cardiovascular outcomes. However, their levels can also be affected by non-cardiac conditions such as renal failure, diabetes, or liver cirrhosis [18,19,20], and their precise role in pediatric arrhythmic disorders, particularly in the absence of structural heart disease, remains incompletely understood (Figure 1) [21].

While the rapid elevation of natriuretic peptides during acute, sustained arrhythmias is well-documented in adult emergency settings, data on baseline neurohormonal profiles in pediatric patients remain remarkably scarce. Most of the clinical evidence focuses on adult cardiomyopathy or structural heart defects, leaving a significant knowledge gap on stable, idiopathic arrhythmias in children and adolescents. It remains unclear whether pediatric patients with a history of these underlying arrhythmias exhibit persistent, subclinical myocardial strain outside of active paroxysms. Therefore, the present preliminary study aimed to determine serum NT-proBNP concentrations in children and adolescents diagnosed with idiopathic ventricular arrhythmias (VAs) or supraventricular tachycardia (SVT) compared to a hospital-based control group. In addition, it evaluated the potential modulating effect of pharmacological antiarrhythmic therapy on NT-proBNP levels. A more refined understanding of this supportive biomarker could lead to a more accurate evaluation of treatment efficacy and help identify pediatric patients at increased risk of acute cardiovascular complications.

## 2. Results

### 2.1. Types of Arrhythmias and Clinical Presentation

The study group consisted of 26 children with a confirmed history of idiopathic cardiac arrhythmias. Ventricular arrhythmias (VAs) occurred in 17 patients (65.38%), including 11 individuals with ventricular extrasystoles (VESs) and six with ventricular tachycardia (VT). Supraventricular arrhythmias (SVAs), presenting as supraventricular tachycardia (SVT), were documented in nine patients (34.62%) (Table 1).

The majority of the patients (23/26) presented with palpitations. Other reported symptoms included chest pain (four cases), weakness (five cases), fainting (twelve cases), dizziness, and shortness of breath. Many patients experienced multiple concurrent symptoms.

An analysis of the three groups of patients with idiopathic cardiac arrhythmias demonstrated diverse paroxysmal dynamics and varying arrhythmic burden profiles. In the supraventricular tachycardia group (SVT, n = 9), the daily arrhythmia burden ranged from 1.1% to 3.1%; for the majority, episodes were infrequent and brief (<45 s), but two patients experienced maximum episode durations of 15 and 30 min. In patients with ventricular tachycardia (VT, n = 6), this burden reached a maximum of 3.5%, predominantly consisting of short non-sustained episodes (NSVT, 3–25 s), though two individuals presented with sustained VT episodes lasting 50 s and 1.5 minutes. The largest cohort, presenting with ventricular ectopy (VES, n = 11), exhibited the widest variance in daily ectopic burden (0.1–30%; ~100 to ~30,000 beats), characterized by prevailing isolated beats and brief ventricular runs (<28 s).

The baseline arrhythmia burden in the study group showed a non-normal distribution, with a median burden of 1.25% (interquartile range [IQR]: 0.00–3.43%, range 0.00–30.00%). The mean arrhythmia burden was 4.40%.

The analysis used serum NT-proBNP concentrations measured by ELISA in pediatric patients with a history of cardiac arrhythmias and non-arrhythmia controls.

### 2.2. Comparison of Study and Control Groups

An initial analysis compared serum NT-proBNP levels between the total group of patients with a history of arrhythmias and the hospital-based controls. Patients diagnosed with cardiac arrhythmias exhibited significantly higher serum concentrations of the biomarker than controls (*p* = 0.03), indicating elevated myocardial strain in the presence of rhythm disturbances (Table 2).

### 2.3. Effect of Pharmacological Treatment

Within the cohort of patients with a history of arrhythmias, a sub-analysis was conducted based on therapeutic status. Treatment-naive patients demonstrated significantly higher levels of biomarkers compared to those on medication. The treated patients exhibited significantly lower serum NT-proBNP concentrations than the untreated individuals (*p* = 0.04). This finding may imply that antiarrhythmic pharmacotherapy has a stabilizing or suppressive effect on peptide release (Table 2).

### 2.4. Comparison by Arrhythmia Type

When evaluating specific arrhythmia subtypes relative to the controls, patients diagnosed with ventricular arrhythmias (VAs) exhibited significantly higher serum NT-proBNP concentrations (*p* = 0.049). Patients with supraventricular tachycardia (SVT) demonstrated an elevated median biomarker level; the difference compared to controls did not reach statistical significance (*p* = 0.15). No statistically significant differences were observed in NT-proBNP levels between the VAs and SVT subgroups (*p* = 0.90), indicating comparable biomarker profiles across different arrhythmia origins (Table 2).

### 2.5. Influence of Sex and Age

As shown in the Study Population section, the study population was well-matched with respect to age and sex. No statistically significant differences in NT-proBNP concentrations were observed between girls and boys (*p* = 0.40). The median value was 5.2 pg/mL (Q1–Q3: 3.7–8.3) in girls and 4.6 pg/mL (Q1–Q3: 3.4–6.6) in boys.

Similarly, a weak and non-significant correlation was found between age and NT-proBNP levels (Spearman’s rank correlation coefficient ρ = 0.10; *p* > 0.05).

### 2.6. Correlation Analysis Using Clinical and Biochemical Parameters

Spearman’s rank correlation coefficients revealed no statistically significant relationships between serum NT-proBNP concentrations and the evaluated echocardiographic parameters (see Table 3). The NT-proBNP levels were not correlated with left ventricular internal diameters in either diastole or systole. No significant relationships were observed regarding left ventricular systolic function, including left ventricular ejection fraction (LVEF) and fraction shortening (FS) (all *p* > 0.05; Table 3).

No significant correlations were found between NT-proBNP levels and routine biochemical or clinical parameters, including creatine kinase (CK), creatine kinase MB isoenzyme (CK-MB), C-reactive protein (CRP), and baseline heart rate across the entire cohort. A distinct intragroup association was identified: in patients with ventricular tachyarrhythmia, a statistically significant negative correlation was observed between serum NT-proBNP concentration and baseline QRS duration during sinus rhythm (Spearman’s rank correlation coefficient ρ = −0.46; *p* < 0.05), indicating a moderate inverse relationship. All other subgroup correlations failed to reach statistical significance (*p* > 0.05).

## 3. Discussion

This pilot study evaluated serum NT-proBNP concentrations in pediatric patients with a history of ventricular and supraventricular tachycardia and assessed the potential effect of pharmacological therapy on this supportive biomarker.

### 3.1. NT-proBNP in Pediatric Arrhythmias

Our results demonstrated significantly higher serum NT-proBNP concentrations in children with a history of arrhythmias compared to hospital-based controls without documented arrhythmias, with the highest values observed in patients in the ventricular arrhythmia (VAs) subgroup. These findings align with the pathophysiological consensus, showing that high natriuretic peptide levels reflect heart strain and hemodynamic burden caused by altered heart rhythms and neurohormonal activation associated with cardiac arrhythmias [28,29,30,31,32,33,34,35]. Importantly, timing of biomarker sampling plays a critical role in interpreting these values. While natriuretic peptides are known to elevate rapidly—within approximately 20 min—from the onset of acute, sustained tachyarrhythmia due to sudden mechanical and hemodynamic stress [36], our findings reflect a baseline neurohormonal profile measured under stable clinical conditions during a diagnostic window. This suggests that pediatric patients with a history of recurrent idiopathic arrhythmias, particularly VAs, may exhibit persistent subclinical myocardial strain even outside of acute arrhythmic episodes [37].

The clinical interpretation of NT-proBNP concentrations in pediatric populations remains challenging due to age-related physiological variability. As previously reported by Gawlik et al. [38], reference ranges for natriuretic peptides in children are relatively limited and highly dependent on complex developmental factors.

Research demonstrates that NT-proBNP concentrations vary progressively with age, exhibiting a sharp decline from the neonatal period through infancy, followed by a gradual stabilization during later childhood and adolescence [39,40,41,42]. Koch and Singer [41], along with Nir et al. [39,42], demonstrated that natriuretic peptide concentrations peak shortly after birth, a trend further supported by Rauch and Koch [40], who highlighted an inverse relationship between age and peptide levels during growth. Saenger et al. [43] and Koerbin et al. [44] suggest that sex-specific reference differences may begin to emerge during adolescence, but no significant associations between NT-proBNP levels and either age or sex were observed in our cohort. This lack of demographic variance is most likely attributable to the relatively narrow and older age distribution of our participants (14–17 years), a developmental window where physiological fluctuations of the peptide stabilize and begin to mirror baseline adult patterns.

### 3.2. NT-proBNP and Arrhythmia Severity

When analyzing specific arrhythmia subtypes, a statistically significant elevation in NT-proBNP was sustained exclusively in the ventricular arrhythmia (VAs) subgroup compared to controls. Although the supraventricular tachycardia (SVT) subgroup displayed an elevated median biomarker value, the difference failed to reach statistical significance, and no significant variation was noted between the VA and SVT cohorts. These localized dynamics partially contrast with findings by Mazurek et al. [22], who observed a more direct escalation of peptide levels scaling with increasing arrhythmic severity phenotypes. In contrast, Zhang et al. [45] and Lin et al. [46] indicated that natriuretic peptide surges correlate tightly with worsening cardiac dysfunction and adverse hemodynamic parameters, while our cohort presented with a completely different clinical profile.

The preserved myocardial structure in our cohort may explain these findings. Kitada et al. [47] demonstrated that a preserved left ventricular ejection fraction (LVEF) is linked to lower, more stable baseline peptide levels compared to states with severely reduced systolic performance. In our study, the cohort had structurally normal hearts with fully preserved systolic function. The absence of correlations between NT-proBNP levels and standard echocardiographic indices (such as LVIDd, LVIDs, LVEF, and LVFS) is compatible with the idiopathic nature of these pediatric arrhythmias. Unlike adult patients, where cardiac arrhythmias frequently stem from underlying ischemic cardiomyopathy, chronic remodeling, or heart failure, our pediatric patients lacked chronic myocardial damage. This absence of structural remodeling may explain why peptide fluctuations did not align with routine echocardiographic parameters, confirming that NT-proBNP release in this specific population reflects a persistent low-grade, neurohormonal response following recent arrhythmia paroxysms, rather than chronic, irreversible myocardial injury or active, acute hemodynamic collapse.

Prolonged episodes of cardiac arrhythmias through both supraventricular and ventricular mechanisms drastically shorten the diastolic phase and impair ventricular filling, thereby inducing severe hemodynamic stress that stimulates the secretion of NT-proBNP. Due to the idiopathic nature of the described arrhythmias (encompassing SVT and ventricular arrhythmia), the rapid elevation of this biomarker reflects a purely dynamic, transient functional overload rather than sustained heart failure or structural myocardial damage. Following arrhythmia conversion and episode termination, natriuretic peptide levels promptly return to the reference values. This process provides biochemical confirmation of the fully reversible nature of the cardiac hemodynamic dysfunction in all studied patient cohorts [23,48,49,50,51].

### 3.3. Pharmacological Treatment and NT-proBNP Levels

One of the most clinically relevant observations of this study was that patients receiving pharmacological treatment exhibited significantly lower NT-proBNP concentrations compared to untreated individuals. A definitive interpretation of this finding is limited by the absence of baseline biomarker measurements prior to therapy initiation. It is widely recognized that cardiovascular agents can modulate natriuretic peptide pathways [52,53,54,55]. Chronic beta-blocker therapy and angiotensin-converting enzyme (ACE) inhibitors have been systematically associated with a reduction in circulating NT-proBNP levels as myocardial workload stabilizes [52,53]. In a large-scale cohort, Kim et al. [55] demonstrated that essential cardiovascular drugs, including beta-blockers, ACE inhibitors, and calcium channel blockers [52,54], exert complex, dose-dependent modulating effects on natriuretic peptide release. In our pediatric cohort, the simultaneous administration of multiple drug classes reflects real-world clinical management but complicates the isolation of individual drug effects [52,53]. Therefore, the observed reduction in NT-proBNP levels in treated patients should be interpreted with caution.

At the same time, magnesium supplementation was utilized in a subset of our patients, and its direct effect on NT-proBNP levels is uncertain. Some studies have found an association between low magnesium levels and increased natriuretic peptide concentrations, as well as a higher cardiovascular risk [56,57]. Kim et al. [55] reported that magnesium oxide administration was associated with an increase in plasma peptide levels. Given the diverse formulations administered in our study (such as magnesium lactate and magnesium hydrogen aspartate tetrahydrate) and the limited sample size, a localized statistical isolation of magnesium’s independent effect was precluded. Similarly, sertraline—administered to a single patient for comorbid minor psychiatric symptoms—exerted no discernible effect on the cardiovascular biomarker, consistent with its non-cardiovascular mechanism of action [58].

### 3.4. Clinical Implications and Electro-Biochemical Interplay

The present findings indicate that NT-proBNP may serve as a potential supportive biomarker of relative myocardial stress in pediatric cardiac arrhythmias. Its utility as a stand-alone tool for differentiating arrhythmia types remains limited. Because absolute NT-proBNP concentrations were lower than those typically reported in pediatric heart failure or structural heart disease, data interpretation should focus on relative differences between groups rather than absolute thresholds.

The absence of correlation between NT-proBNP and echocardiographic indices reflects the idiopathic nature of these pediatric arrhythmias. While correlations with ventricular remodeling indices are well-documented in structural heart defects or cardiomyopathies [59,60], the preserved myocardial architecture in idiopathic substrates precludes such chronic biophysical adjustments.

Our subgroup analysis revealed a physiological interplay: a moderate and statistically significant negative correlation between NT-proBNP and baseline QRS in the VAs cohort. Because these patients have structurally normal hearts, their baseline QRS complexes during sinus rhythm are characteristically narrow, as frequently observed in pediatric substrates presenting with ventricular arrhythmias (VAs), such as right ventricular outflow tract ventricular tachycardia (RVOT-VT) [61,62].

The absence of baseline conduction system fibrosis means that the baseline elevation of NT-proBNP observed in our cohort likely represents a persistent neurohormonal signature of recent tachycardia paroxysms, during which rapid peptide release was triggered by acute hemodynamic overload and wall stretch [23], rather than chronic structural damage [33]. This is compatible with stretch-induced arrhythmia mediated by mechano-electric feedback [63,64].

Antiarrhythmic therapy likely contributed to this paradoxical trend. Clinically stable patients with lower levels of NT-proBNP were eligible for propafenone treatment. As a potent class Ic sodium-channel blocker, it slows intraventricular conduction and prolongs baseline QRS duration. This agent was also restricted in more acute cases with higher hemodynamic stress (elevated NT-proBNP), leaving them with shorter, unaltered QRS complexes. This therapeutic distribution accentuates the negative correlation within an otherwise healthy myocardial substrate [61,62]. For the clinician, this underscores that a narrower baseline QRS complex in an adolescent with VAs does not imply lower hemodynamic stress. Combining electrocardiographic conduction parameters with ambulatory NT-proBNP monitoring may offer a more nuanced, individualized approach to therapeutic monitoring in pediatric idiopathic arrhythmias.

### 3.5. Study Limitations

Several limitations must be acknowledged when interpreting these results. This pilot study had a limited sample size, which restricted statistical power and precluded the use of multivariate regression analysis or adjustments for multiple comparisons. Consequently, the findings must be viewed as exploratory and hypothesis-generating.

The timing of blood sampling represents another important methodological concern. Peripheral blood samples were obtained under stable clinical conditions rather than during acute, active tachyarrhythmia episodes. This should be taken into account when comparing our results with data obtained during acute arrhythmic episodes. In addition, a detailed electrophysiological breakdown into specific arrhythmia sub-mechanisms, such as distinguishing ventricular extrasystoles from sustained ventricular tachycardia or atrioventricular nodal reentrant tachycardia (AVNRT) versus atrioventricular reentrant tachycardia (AVRT), and the exact tracking of paroxysm cumulative duration were not feasible due to the non-invasive nature of this pilot study, which may limit the isolation of specific subtype-dependent hemodynamic effects on NT-proBNP levels.

The absence of serial baseline biomarker measurements captured immediately prior to the initiation of pharmacotherapy limits our ability to definitively establish a causal relationship between drug use and the subsequent reduction in NT-proBNP levels.

No long-term endpoints of clinical outcome or recurrence rates were examined, which restricts the prognostic interpretation of the evaluated peptide dynamics. Future prospective multicenter studies with larger pediatric cohorts are warranted to validate these relationships.

### 3.6. Summary

Despite these limitations, this study provides preliminary evidence that serum NT-proBNP concentrations are elevated in pediatric patients with a history of arrhythmias, particularly those with ventricular arrhythmias (VAs). These findings may indicate the potential role of NT-proBNP as a supportive biomarker of myocardial strain in this clinical population. Further large-scale prospective studies are required to validate these relationships fully.

## 4. Materials and Methods

### 4.1. Study Population

Demographic and clinical data for both the hospital-based controls (8 boys and 12 girls admitted for the diagnostic evaluation of atypical chest pain or vasovagal syncope, without documented arrhythmias) and the cohort of patients with a history of cardiac arrhythmias (12 boys and 14 girls) were obtained from the Department of Pediatric Cardiology at the Upper Silesian Children’s Health Center in Katowice, Poland.

The inclusion criteria for the study group were as follows: age 14–17 years; a confirmed diagnosis of idiopathic supraventricular tachycardia (SVT) or ventricular arrhythmias (VAs); a documented arrhythmia burden on 24 h ambulatory Holter electrocardiography (ECG) monitoring; and written informed consent to participate. The exclusion criteria were age under 14 years; postoperative, congenital, or inflammatory cardiac arrhythmias; and structural heart disease. Patients with a history of prior catheter ablation or those who underwent the procedure during the study period were also excluded from the analysis.

Consequently, a total of 26 children and adolescents were enrolled in the arrhythmia group, comprising patients with SVT (n = 9) and VAs (n = 17). All arrhythmias in this cohort were confirmed as idiopathic, with no underlying structural or congenital heart disease detected by comprehensive transthoracic echocardiography (TTE). All participants underwent a detailed clinical evaluation, including medical history assessment, physical examination, routine laboratory tests, and resting blood pressure measurements, with particular emphasis on potential arrhythmogenic triggers. Additionally, 24 h ambulatory Holter ECG monitoring, treadmill exercise testing, and body mass index (BMI) assessments were performed.

The controls and study groups were well-matched with respect to demographic and anthropometric characteristics. The mean age was 15.6 ± 1.2 years in the control group and 15.6 ± 1.8 years in the arrhythmia group. No statistically significant differences were observed between the groups regarding height (168.9 ± 8.7 cm vs. 167.8 ± 5.9 cm; *p* = 0.64), body weight (61.9 ± 16.2 kg vs. 64.8 ± 12.8 kg; *p* = 0.50), body surface area (1.70 ± 0.21 m^2^ vs. 1.73 ± 0.17 m^2^; *p* = 0.60), or BMI (21.6 ± 5.3 kg/m^2^ vs. 22.8 ± 4.0 kg/m^2^; *p* = 0.40) (Table 4).

The close matching of the study and control cohorts minimized the potential influence of confounding anthropometric variables. In pediatric research, achieving such strict comparability is highly desirable due to the dynamic physiological and hemodynamic changes associated with pubertal growth and development.

Potential confounding cardiac and systemic factors were further evaluated through laboratory analyses, including creatine kinase (CK), creatine kinase MB isoenzyme (CK-MB), C-reactive protein (CRP), high-sensitivity cardiac troponin, third-generation thyroid-stimulating hormone (TSH), and free thyroxine (FT4). No statistically significant differences in these biochemical parameters were observed between patients with a history of cardiac arrhythmias and the hospital-based controls.

This study represents a continuation of our ongoing institutional research program entitled “Epigenetic, Pharmacogenetic, and Molecular Aspects of Arrhythmogenic Heart Disease in Children and Adolescents” [65,66,67].

At the time of blood collection, eleven patients with a confirmed history of arrhythmia were treatment-naive. The remaining participants with an arrhythmia history received stable pharmacological regimens that could potentially modulate cardiovascular hemodynamics and influence NT-proBNP concentrations. These medications included β-blockers (metoprolol tartrate, metoprolol succinate, bisoprolol fumarate, sotalol, and propranolol), angiotensin-converting enzyme (ACE) inhibitors (enalapril maleate), sodium-channel blockers (propafenone hydrochloride), and sertraline, a selective serotonin reuptake inhibitor prescribed for comorbid minor depressive or anxiety symptoms (Table 5). No antiarrhythmic or cardiovascular medications were used in the control group.

### 4.2. Serum Collection and Storage

Peripheral blood samples were collected at the Department of Pediatric Cardiology, Upper Silesian Children’s Health Center. Blood was drawn into serum separation tubes and allowed to clot completely at room temperature for 2 h. Following clotting, the samples were immediately processed by centrifugation at 1000× *g* for 20 min at room temperature. The separated serum aliquots were immediately frozen and stored at −80 °C until biochemical analysis. All samples were obtained under stable clinical conditions, during elective hospitalization (either on the day of admission or the following day), without acute hemodynamic interventions, and within six months of the initial clinical diagnosis of the underlying arrhythmia.

### 4.3. Ethical Considerations

The study protocol was reviewed and approved by the Bioethics Committee of the Medical University of Silesia (approval number: PCN/CBN/0052/KB1/142/I/17/18/22). Written informed consent was obtained from the parents or legal guardians, alongside written consent from the minor participants themselves prior to study enrollment. The study was conducted in strict accordance with the ethical principles outlined in the Declaration of Helsinki and relevant national regulations governing research involving human participants [65,66,67].

### 4.4. Measurement of NT-proBNP Concentrations

Serum NT-proBNP concentrations were measured using a commercially available enzyme-linked immunosorbent assay (ELISA) kit specific for human N-terminal pro-B-type natriuretic peptide (Cloud-Clone Corp., Katy, TX, USA). The frozen serum aliquots were rapidly thawed and allowed to equilibrate to room temperature immediately prior to analysis. All assays were performed in duplicate in strict accordance with the manufacturer’s specified protocol.

Absorbance was measured using a Labtech LT-5000 microplate reader (Labtech International, Heathfield, UK) at an optical density (OD) of 450 nm. NT-proBNP concentrations were calculated mathematically based on the generated standard curve and expressed as picograms per milliliter (pg/mL).

### 4.5. Statistical Analysis

Statistical analyses were performed using Statistica version 13.3 (TIBCO Software Inc., Palo Alto, CA, USA). The normality of data distribution was evaluated using the Shapiro–Wilk test. The results concerning clinical parameters (Table 4) reflected a normal distribution; therefore, the parametric Student’s *t*-test was used in the statistical analysis. These data are expressed as the mean and standard deviation (SD). Continuous variables with non-normal distributions are expressed as medians with interquartile ranges (IQRs; lower and upper quartiles, Q1–Q3). Continuous data between the two independent groups were compared using the non-parametric Mann–Whitney U test (Table 2). Relationships between continuous variables were assessed using Spearman’s rank correlation coefficient ρ. A two-sided *p*-value < 0.05 was considered statistically significant. Due to sample size constraints, multivariate regression analysis was not performed, and no adjustment for multiple comparisons was applied.

## 5. Conclusions

This pilot study provides preliminary evidence that serum NT-proBNP concentrations are significantly elevated in pediatric patients with a history of cardiac arrhythmias compared to hospital-based controls without confirmed rhythm disturbances, reflecting a state of relative myocardial strain. This increase was particularly evident within the ventricular arrhythmia (VA) subgroup relative to controls; no statistically significant differences in biomarker levels were observed between the VA and supraventricular tachycardia (SVT) subgroups. Within this adolescent cohort, serum NT-proBNP concentrations were not significantly affected by demographic factors, showing no meaningful associations with age or sex.

Significantly lower NT-proBNP concentrations were observed in patients receiving pharmacological treatment compared to untreated individuals. While this suggests a potential stabilizing effect of antiarrhythmic therapy, a definitive causal interpretation was precluded due to the heterogeneity of treatment regimens and the absence of baseline, pre-treatment biomarker measurements.

In conclusion, these findings may indicate the potential usefulness of NT-proBNP as a supportive biomarker of myocardial strain in pediatric patients with a history of these underlying arrhythmias. However, given its overlapping profiles across arrhythmia types and the lack of correlation with routine echocardiographic parameters, it should not be utilized as a stand-alone diagnostic tool for arrhythmia classification or clinical risk stratification. Future large-scale, prospective multicenter studies with standardized therapeutic protocols and longitudinal biomarker assessments are required to fully clarify its prognostic and clinical utility in pediatric patients.

## Figures and Tables

**Table 1:** Detailed breakdown of specific cardiac arrhythmia subtypes in the study group.

Type of Arrhythmia	Arrhythmia Subtype	Number of Patients (n)	Percentage (%)
Ventricular (65.38%)	Ventricular extrasystoles (VESs)	11	42.31%
	Ventricular tachycardia (VT)	6	23.07%
Supraventricular (34.62%)	Supraventricular tachycardia (SVT)	9	34.62%
Total (100.00%)		26	100.00%

**Abbreviations:** VESs—ventricular extrasystoles; VT—ventricular tachycardia; SVT—Supraventricular tachycardia.

**Table 2:** Comparison of serum NT-proBNP concentrations (pg/mL) between the study and control groups, with sub-analyses based on clinical presentation and treatment status.

Analyzed Subgroups	Median (Me)	95% CI for Median	Minimum	Maximum	IQR	*p*-Value
**Arrhythmia Patients** (n = 26)	5.77	3.92–14.82	2.43	72.84	3.53	
**Controls** (n = 20)	3.98	3.79–6.23	2.75	14.14	2.43	***p* = 0.03**
**Treated Patients** (n = 15)	5.25	4.06–7.89	2.43	14.14	2.85	
**Untreated Patients** (n = 11)	6.90	3.87–16.61	3.87	72.84	9.36	***p* = 0.04**
**Controls** (n = 20)	3.98	3.79–6.23	2.75	14.14	2.43	
**SVT Patients** (n = 9)	6.32	3.95–9.12	2.43	13.68	2.60	*p* = 0.15
**Controls** (n = 20)	3.98	3.79–6.23	2.75	14.14	2.43	
**VAs Patients** (n = 17)	5.77	2.39–19.35	2.92	72.84	2.83	***p* = 0.049**
**SVT Patients** (n = 9)	6.32	3.95–9.12	2.43	13.68	2.60	
**VAs Patients** (n = 17)	5.77	2.39–19.35	2.92	72.84	2.83	*p* = 0.90

**Abbreviations:** CI, confidence interval; IQR, interquartile range; SVT, supraventricular tachycardia; VAs, ventricular arrhythmias. Statistically significant *p*-values (*p* < 0.05) are given in bold.

**Table 3:** Spearman’s rank correlation coefficients for NT-proBNP and echocardiographic parameters.

Variable	NTproBNP	LVIDd	LVIDs	LVEF	LVFS
**NT-proBNP**	1.000	0.059	0.058	−0.023	−0.136
***p*-value**	>0.05	>0.05	>0.05	>0.05	>0.05

**Abbreviations:** LVIDd, left ventricular internal diameter at end diastole; LVIDs, left ventricular internal diameter at end systole; LVEF, left ventricular ejection fraction; LVFS, left ventricular fraction shortening. Pairwise deletion was applied for missing data. Statistically significant correlations are defined at *p* < 0.05 (no significant correlations were observed).

**Table 4:** General characteristics of patients with arrhythmias and controls.

General Characteristics	Study Group (n = 26)	Controls (n = 20)	*p*-Value
Gender (F/M)	14/12	12/8	0.68
Mean age (years)	15.6 ± 1.8	15.8 ± 1.2	0.86
Height (cm)	168.9 ± 8.7	167.8 ± 5.9	0.64
Weight (kg)	61.9 ± 16.2	64.8 ± 12.8	0.50
BSA [m^2^]	1.70 ± 0.21	1.73 ± 0.17	0.60
BMI	21.6 ± 5.3	22.8 ± 4.0	0.40
BP syst (mmHg)	116.9 ± 10.8	120.5 ± 14.1	0.35
BP diast (mmHg)	64.3 ± 8.5	63.0 ± 10.5	0.66
Sat O_2_ (%)	98.3 ± 0.7	98.4 ± 1.1	0.75
** Laboratory Tests**			
CK (U/L)	75.4 ± 16.9	95.3 ± 54.8	0.20
CK-MB (U/L)	13.4 ± 5.3	12.8 ± 5.7	0.76
CRP (mg/L)	2.3 ± 3.9	2.9 ± 6.4	0.80
WBCs (10^3^ U/L)	6.7 ± 1.85	6.82 ± 2.04	0.83
RBCs (10^6^ U/L)	4.84 ± 0.47	4.81 ± 0.45	0.81
HGB (g/dL)	13.74 ± 1.24	14.1 ± 1.28	0.42
HCT (%)	41.3 ± 3.5	42.2 ± 3.8	0.40
TSH (mL U/L)	2.52 ± 1.07	2.39 ± 1.23	0.73
FT4 (ng/dL)	1.3 ± 0.18	1.4 ± 0.39	0.43
** Holter ECG Findings**			
Number of QRS complex/24 h	99,448 ± 15,715	97,065 ± 10,124	0.54
Abnormal QRS mean (%) min–max	4.4 (1–30)	0	n/a
HR mean	74.4 ± 11.5	72.2 ± 7.9	0.45
HR max	126.2 ± 18.8	124.3 ± 15.2	0.70
HR min	55.6 ± 8.2	52.9 ± 6.4	0.40
** Echocardiography Parameters**			
LVIDd (mm)	48.4 ± 4.5	49.0 ± 3.7	0.79
LVIDs (mm)	29.3 ± 4.5	29.1 ± 3.3	0.79
LVEF (%)	68.3 ± 8.5	70.0 ± 5.8	0.40
LVFS (%)	40.5 ± 6.4	40.0 ± 4.8	0.76
** Exercise Test**			
Stress test (gr)	3.7 ± 0.7	3.8 ± 0.5	0.60
MET	11.3 ± 1.2	11.5 ± 0.72	0.48
NYHA Class (I/II)	20/6	18/2	0.25

**Abbreviations**: BSA, body surface area; BMI, body mass index; BP syst/diast, systolic and diastolic blood pressure; Sat O_2_ (%), oxygen saturation; CK, creatine kinase; CK-MB, creatine kinase MB fraction; CRP, C-reactive protein; WBCs, white blood cells (leukocytes); RBCs, red blood cells (erythrocytes); HGB, hemoglobin; HCT, hematocrit; TSH, thyroid hormone; FT4, thyroxine; ECG, electrocardiogram; QRS, QRS interval in electrocardiogram (Q wave, R wave, and S wave in ECG); HR, heart rate; LVIDd, left ventricular internal diameter at end diastole; LVIDs, left ventricular internal diameter at end systole; LVEF, left ventricle ejection fraction; LVFS, left ventricular fraction shortening; MET, metabolic equivalents of task; NYHA Class, New York Heart Association Heart Failure Classification: Class I—no symptoms and no limitation in ordinary physical activity, e.g., shortness of breath when walking, climbing stairs, etc. Class II—mild symptoms (mild shortness of breath and/or angina) and slight limitation during ordinary activity.

**Table 5:** Pharmacotherapy in the study group (n = 26).

Drug Class	Active Substance(s)	Number of Patients
β-blockers	Metoprolol, bisoprolol, propranolol, sotalol	12
ACE inhibitors	Enalapril	1
Sodium-channel blockers	Propafenone	5
Magnesium supplementation	magnesium lactate, magnesium aspartate	13
SSRI	Sertraline	1
No pharmacotherapy	-	11

**Abbreviations:** ACE, angiotensin-converting enzyme; SSRI, selective serotonin reuptake inhibitor. The total number of patients across categories exceeds (n = 26) due to combination therapy in some individuals. No antiarrhythmic or cardiovascular medications were used in the control group.

## Data Availability

The data are unavailable due to privacy and ethical restrictions. The data generated in this study are available from the corresponding authors upon reasonable request.

## Abbreviations

The following abbreviations are used in this manuscript:

NT-ProBNP	N-terminal pro-B-type natriuretic peptide
SCD	sudden cardiac death
ECG	electrocardiogram
BNP	B-type natriuretic peptide
RAA system	renin–angiotensin–aldosterone system
VT	ventricular tachycardia
SVT	supraventricular tachycardia
BMI	body mass index
CK	creatine kinase
CK-MB	muscle isoenzyme of the CK
CRP	C-reactive protein
TSH	thyroid-stimulating hormone
FT4	thyroxine
ELISA	enzyme-linked immunosorbent assay method
LVIDd	left ventricular internal diameter at end diastole
LVIDs	left ventricular internal diameter at end systole
LVEF	left ventricular ejection fraction
LVFS	left ventricular fraction shortening
QRS complex	QRS interval in ECG
HR	heart rate
ACE	angiotensin-converting enzyme
CI	confidence interval
